# Advances in Human Metapneumovirus Research: Clinical Impact, Diagnostic Innovations, and Therapeutic Challenges

**DOI:** 10.3390/pathogens14121277

**Published:** 2025-12-12

**Authors:** Md Mostafizur Rahman, Parul Suri, Mimnu Tasnim, Moushumi Afroza Mou, Amatun Noor Prapty, Rakhee Rathnam Kalari Kandy

**Affiliations:** 1 Laboratory of RNA Biology and Molecular Neuroscience, Department of Biological Sciences, St. John’s University, Queens, New York, NY 11432, USA; 2 Department of Surgery, Kurmitola General Hospital, Dhaka 1206, Bangladesh; 3 Department of Biotechnology, Jaypee Institute of Information Technology, Noida 201309, India; 4 Family Medicine, Efficient Medical Care PC, Queens, New York, NY 11432, USA; tasnim.mimnu@gmail.com; 5 Laboratory of Molecular Physiology and Biophysics, Department of Biological Sciences, St. John’s University, Queens, New York, NY 11432, USA; 6 Critical Care Medicine, University of Texas MD Anderson Cancer Center, Houston, TX 77030, USA

**Keywords:** human metapneumovirus, hMPV, respiratory tract infection, diagnostics, RT-PCR, monoclonal antibodies, vaccine development, fusion protein

## Abstract

Since its discovery in 2001, Human Metapneumovirus (hMPV) has been identified globally, exhibiting predictable seasonal outbreaks primarily affecting young children, the elderly, and individuals with preexisting health conditions. The virus is transmitted through airborne droplets and is responsible for a notable percentage of respiratory illnesses, particularly in children under five years of age, with hospitalization rates peaking in the first year of life. The complex immune response elicited by hMPV, characterized by a Th17-like profile and excessive mucus production, contributes to respiratory complications, emphasizing the need for effective management strategies. This review discusses various diagnostic methods, emphasizing the potential of combining serology with RT-PCR to enhance diagnostic accuracy during outbreaks. Furthermore, it addresses the therapeutic approaches, including the promise of recombinant interferons and ongoing research on the use of passive immunity through neutralizing antibodies. A comprehensive overview of hMPV, emphasizing the importance of continued research to improve diagnostic and therapeutic options for this significant respiratory pathogen, offers promising strategies for manipulating responses through targeted interventions.

## 1. Introduction

Human Metapneumovirus (hMPV) was initially identified by van Den Hoogen et al., 2001, in Dutch children with acute lower respiratory tract infection (LRTI) [1]. The pathology and clinical symptoms exhibited by hMPV are similar to those of the previously reported human respiratory syncytial virus (RSV) [2]. Clinically, hMPV infections are manifested by upper and lower respiratory tract infections, where LRTIs predominate with symptoms like bronchitis, bronchiolitis, cough, and other respiratory complications developed due to the absence of airflow [2,3,4]. Belonging to the *Pneumoviridae* family and *Metapneumovirus* genus, hMPV is an enveloped, negative-sense, single-stranded RNA virus that shares many genomic and clinical features with RSV. However, it lacks the nonstructural genes NS1 and NS2 [5]. hMPV infections span all age groups but pose the highest risk to infants, elderly adults, individuals with underlying cardiopulmonary conditions, and immunocompromised patients, including hematopoietic stem cell transplant recipients [6]. Furthermore, hMPV infection in children under 2 years of age could lead to the development of asthma in the later stage of their life, and it also promotes chronic obstructive pulmonary disease (COPD) [2,7,8,9]. hMPV infection has been reported to cause 10–12% of virus-associated respiratory illness and hospitalization in children [4], and this infection is equivalent to influenza virus and parainfluenza virus types 1–3 [10].

hMPV infection results in a Th17-like immune response characterized by interleukin-6 (IL-6) and TNF⍺ secretion in the lungs, followed by an inadequate Th2-like profile resulting in the early secretion of various proinflammatory cytokines such as IL-4, IL-5, and IL-8 [11,12,13,14]. Mice model studies showed that a cytokine, thymic stromal lymphopoietin (TSLP), causes a delay in Th1-like immune response, ultimately stimulating cytokines related to a Th2-like profile with increased neutrophil infiltration upon infection [15]. This complex immune response and excessive mucus production collapse the respiratory airways [3,7]. Although hMPV is now globally distributed, its true burden remains underestimated due to limited testing, especially in low-resource settings. Historically, diagnosis relied on viral culture and serology, which lacked sensitivity and timeliness. However, the integration of molecular diagnostics, particularly real-time reverse transcription PCR (RT-PCR) and multiplex respiratory panels, has significantly improved case detection and epidemiological tracking [16].

**Figure 1:**
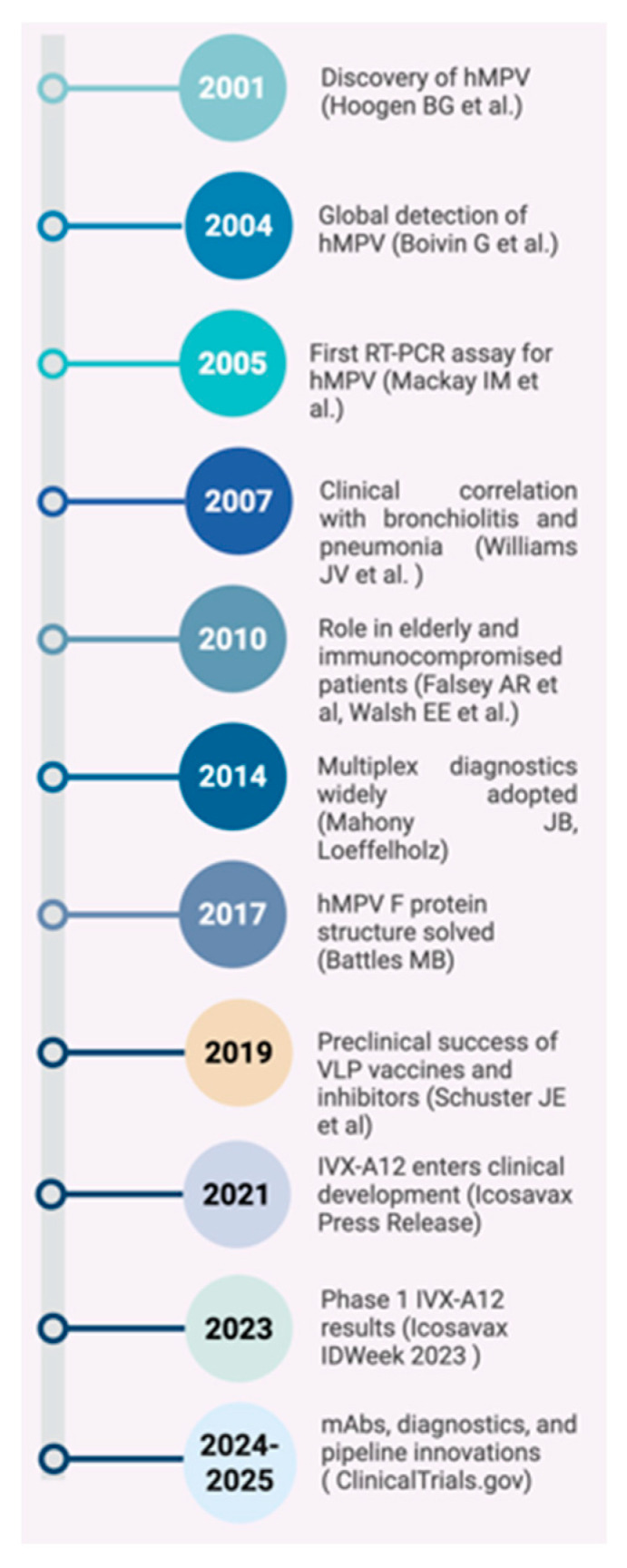
Milestones in Human Metapneumovirus Research: From Discovery to Vaccine Development (2001–2025) [1,16,19,20,21,22,23,24].

Despite improved diagnostics, no approved antiviral therapies or licensed vaccines for hMPV are currently available [Figure 1]. Supportive care remains the cornerstone of treatment, and most infections are self-limiting. Nevertheless, in severe cases, especially among high-risk populations, complications can lead to hospitalization and increased healthcare utilization [17]. Recent preclinical and clinical advancements show promise, including the development of virus-like particle (VLP)-based vaccines such as IVX-A12, monoclonal antibodies targeting the F protein, and small-molecule fusion inhibitors [18]. Understanding hMPV’s immune evasion mechanisms, host–pathogen interactions, and cross-protective immunity is pivotal for advancing therapeutic and preventive strategies. This review critically examines recent advances in the understanding of hMPV clinical impact across age and risk groups, evaluates current and emerging diagnostic strategies, and analyzes the therapeutic and vaccine development pipeline with emphasis on remaining challenges and priorities.

## 2. Materials and Methods—Literature Search Strategy

We conducted a narrative search using multiple keywords including “hMPV”, “diagnostics”, “treatment” and “therapeutics” on PubMed, Scopus and Google Scholar databases to look for research papers, review articles, guidelines, and studies to obtain relevant information, limited to studies published in the last 25 years (2001–2025). The analysis only included research articles, studies, guidelines, and review papers that were obtainable in English. Research that (i) was not written in English, (ii) comprised of comments, letters, case reports, abstracts from conferences, or (iii) had multiple entries were not included.

## 3. Epidemiology

**Figure 2:**
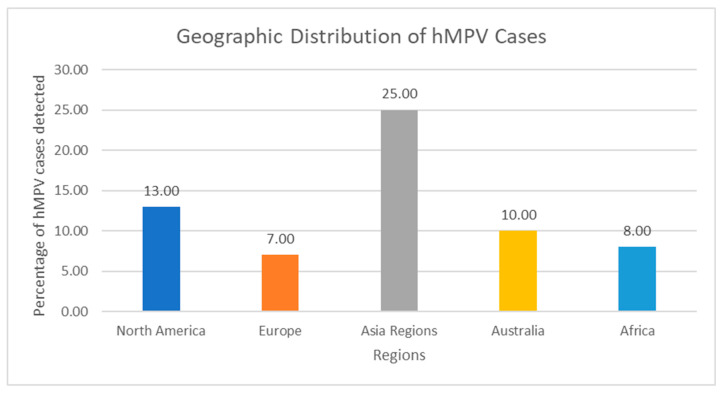
Geographic Distribution of hMPV Cases. Bar Chart: X-axis: Regions; Y-axis: Percentage of hMPV detection in acute respiratory illness—North America: 6–13%, Europe: 5–12%, Asia: 7–43%, Latin America: 3–15%, Africa: 4–18%, Australia: 7–10%. Note: These are not results of standardized global surveillance. Prevalence varies markedly according to testing practices, case definitions, seasonality, and inclusion of community vs. hospital cases. Most low- and middle-income regions (large parts of Latin America, sub-Saharan Africa, South/Southeast Asia) remain under-represented because routine multiplex panels are rarely used. The erroneous assignment of Italian data to “Asia” in the previous version has been corrected.

hMPV has been discovered in virtually all regions of the world since its first description in 2001, with documented circulation on every inhabited continent, including extensive reports from Latin America (Brazil, Argentina, Chile, Peru, Colombia, Mexico), the Middle East, and multiple sub-Saharan African countries. Considering observations from North America (the United States and Canada), Europe (the United Kingdom, France, Germany, Italy, Spain, and Finland), Asia (Hong Kong and Japan), and Australia, hMPV has been discovered in the majority of regions of the world [25] [Figure 2]. hMPV has a predictable seasonality and has been detected on every continent. hMPV spreads by infectious airborne droplets like other respiratory viruses. It primarily affects young children and newborns, the elderly, and people with preexisting chronic illnesses, including emphysema, asthma, or compromised immune systems [1,26]. HIV-positive and non-immunocompromised South African children have also been shown to carry the virus [27].

The majority of children globally acquire infection with hMPV by the time they are 5 years old, according to sero-epidemiology research [28,29,30,31,32]. Nevertheless, reinfection happens, particularly in older and high-risk individuals, as a result of immune reactions that are not fully protective or infection with a novel genotype [30,33]. Cynomolgus macaques have a temporary immune defense to experimental hMPV infection, as shown by Van den Hoogen et al. [34]. Over the cold season of 2000–2001, Boivin et al. discovered hMPV in 2.3% of breath specimens [3]. Among the 26 hospitalized patients infected with hMPV, 35% were younger than 5 years old, and 46% were older than 65. A preexisting medical condition was present in one-third of hospitalized children below five years old, two-thirds of patients between the ages of 15 and 65, and all patients over 65 [35]. Although they happen across infancy, hospitalization rates for hMPV disease are greatest in the initial year of life. Hospitalization for hMPV peaks from 6 to 12 months of age, which is later than the peak for RSV (2–3 months), according to numerous studies [3,10,36,37,38,39,40]. According to the scant seroprevalence assessments conducted in Israel [29], Japan [28], and The Netherlands [1], nearly every child gets the disease by the time they are 5 to 10 years old.

Since its discovery in 2001, hMPV has been retrospectively and prospectively identified in virtually all regions where systematic testing has been performed. However, the reported detection rates vary widely (2–43% of acute respiratory infections) because of differences in study design, population, season, and diagnostic methods [3].

Pre-2015 era (mostly singleplex RT-PCR or culture-based hospital studies): detection rates in hospitalized children were typically 5–15% [3,10,36] and 2–13%in adults and elderly [35]. These figures almost certainly underestimated the true contribution because testing was restricted to RSV/influenza-negative samples.

2015–2025 era (widespread adoption of commercial multiplex panels): now report hMPV in 4–12% of all community-acquired respiratory illnesses across all ages, with peaks of 15–25% in children <5 years during winter–spring months in temperate climates [37,38]. In tropical regions the seasonality is less pronounced or bimodal.

High-risk groups (2018–2025 data): among hematopoietic stem-cell or solid-organ transplant recipients, hMPV accounts for 3–12% of respiratory viral infections, with progression to lower respiratory tract disease in 30–80% of cases [30,33].

Many fewer studies have been conducted on the relative contribution of hMPV to adult respiratory disorders. As much as 13 percent of hospitalized adults in Rochester, New York, have hMPV [33]. hMPV infection causes higher severity of illness and high rates of illness and death in the elderly, although it is usually moderate in normally well younger people. According to a later study, bronchitis or pneumonia struck at least 50% of older people infected with hMPV during an outbreak in a long-term care facility, resulting in a 50% fatality rate [19]. Over the course of four successive winters, Walsh et al. discovered that the percentage of adult hMPV infections ranged from 3% to 7.1% [33]. This is higher compared to the influenza A infection rate of 2.4% in identical groups over the same period of time and comparable to the yearly average infection rate for RSV (5.5%) [20]. A total of 2.2% of patients with community-transmitted acute RTI who saw a general physician and tested negative for influenza virus and RSV had hMPV [21]. According to Widmer et al., hMPV was responsible for 4.5% of acute RTI hospitalizations in persons over 50 during the colder months of three consecutive years [41]. RSV and influenza A had corresponding rates of 6.1% and 6.5%. The average yearly hospitalization rates for hMPV were 22.1/10,000 residents for people over 65 and 1.8/10,000 residents for persons aged 50–65. Relative to those diagnosed with influenza, those with hMPV infections were older, more prone to cardiovascular illness, and more inclined to receive an influenza vaccination [35].

Factors that underlie the severity of the illness and hospitalization are influenced by pre-existing illnesses, especially asthma. Seven percent of adults hospitalized for an acute asthma attack had hMPV isolated from them [22]. Similar to RSV infection, hMPV infection during the first two years of infancy increases the risk of developing asthma later in life [8]. According to one study, 16% of those diagnosed with hMPV had asthma, but not one of the 54 RSV+ individuals had this condition [38]. A total of 41% of hMPV+ children aged 5 to 13 had a prior asthma diagnosis, according to a separate study [23]. Individuals with preexisting medical disorders and those with impaired immune systems may be particularly vulnerable to hMPV. According to a study, a large number of hospitalized hMPV + patients older than five had other serious illnesses, including lymphoma or cystic fibrosis [38]. According to a different retrospective study, four of the 39 immunocompromised children with hMPV died from respiratory failure, and 17 of them developed pneumonia [42]. Sixty-seven percent of a group of patients aged 15 to 65 had underlying illnesses, including lung tumors or lymphoma [17]. Co-infections with additional bacterial or viral pathogens might make symptoms worse. Individuals with hMPV have viral co-infection frequencies ranging from 6 to 23% [23,33,43]; however, the severity of the disease appears to be unaffected by viral co-infections [43]. Recurrent bacterial pneumonia, on the other hand, is possible and linked to a higher death rate [44,45,46].

## 4. Structure and Function of hMPV

hMPV is a member of the enveloped, negative-sense, single-stranded RNA virus family Pneumoviridae and is closely linked to the Avian metapneumovirus (AMPV) [25]. hMPV diverged from AMPV, most especially subtype C, approximately 200–300 years ago [47,48,49,50,51]. It encompasses two main genetic lineages, A and B, of which A1, A2 (including A2a and A2b), and B1, B2 sublineages are further differentiated [5]. Lineage A2 is more common worldwide and is linked to severe respiratory illnesses [52].

Similar in structure to other Pneumoviridae, hMPV has a 150–200 nm diameter and a spherical to pleomorphic morphology. Surface glycoproteins necessary for entry and immune evasion are embedded in the virus’s outer lipid envelope, produced from the host cell membrane [53].

Nucleoprotein (N), Phosphoprotein (P), Matrix protein (M), Fusion protein (F), Attachment glycoprotein (G), Small hydrophobic protein (SH), Large polymerase protein (L), and M2 (accessory) protein are the primary proteins encoded by the hMPV genome, which is about 13.3 kilobase (kb). The gene sequence is 3’-N-P-M-F-M2-SH-G-L-5’ [54].

G protein shows redundancy with the F protein by aiding adhesion to host cell receptors, but it is not necessary for replication in vitro [55]. Additionally, there is strong evidence that the G protein helps to suppress the interferon-I (IFN-I) response [56], and it also helps to draw neutrophils to the airways by releasing more of the chemoattractants such as CXCL2, CCL3, CCL4, IL-17, and TNF [57]. In addition, SH protein may also operate as a viroporin and plays many roles in modulating the innate immune response, including inhibiting the IFN response [58]. Although its exact function is unknown, it is thought to influence host immune responses by preventing apoptosis. The G and SH proteins decrease the generation of type I interferon by interfering with pattern recognition receptor (PRR) signaling [59].

F protein mediates membrane fusion to enable viral entry. A fusion peptide is revealed by proteolytic cleavage, which is essential for infectivity [60]. Moreover, L protein, the large polymerase protein, can create new genetic material with cofactors. It has zinc-binding sites and diverse catalytic activity [61]. Matrix (M) protein connects the nucleocapsid to the lipid envelope and is essential for virion formation and budding. It has a Ca^2+^ binding site with high affinity [62]. This protein can cause the release of inflammatory cytokines in vitro cultures and appears to be secreted by infected cells in a soluble form [63]. The N protein builds the nucleocapsid, which firmly encloses the RNA genome to prevent its destruction. The N protein suppresses the release of pro-inflammatory cytokines by blocking the activation of the nuclear factor kappa-light-chain-enhancer of activated B cells (NF-κB) [64]. To facilitate transcription and replication, the P protein serves as a cofactor for the L protein (RNA-dependent RNA polymerase) [65]. The inclusion bodies commonly detected during hMPV infections are formed mainly by these two proteins, N and P [66].

## 5. Receptor Mechanism of hMPV Infection

Human and mouse model studies confirmed that the upper and lower respiratory tract and the lung resident leukocytes were the primary targets of hMPV [67]. Upon infection, hMPV causes a series of histopathological changes in the infected lungs that include loss of ciliation, damage to the respiratory epithelial architecture, exacerbated mucus production, sloughing of epithelial cells, and parenchymal pneumonia (inflammation of the lung interstitium) [7,68]. However, the exact mechanism behind hMPV immune evasion has not been clearly understood. The airway epithelial cell (AEC) infection is the first critical stage of acute hMPV infection [69]. In addition, airway macrophages and dendritic cells (DCs) also play a vital role in sensing hMPV infection and its further development into airway inflammation [15,70].

Viral infections commonly trigger innate and adaptive immune responses, primarily attributed to the upregulated expression of IFN, and it is further mediated by the downstream interferon-stimulated genes (ISGs) [71]. This, in turn, triggers and activates the interferon receptors (IFNR) expressed on the target cells, leading to antigen-dependent and independent cytotoxicity and ultimately eliminating the pathogen [71,72,73]. The following mechanisms are now considered well established because they have been independently reproduced by several laboratories using primary human airway epithelial cultures, murine models, and non-human primates. Studies revealed two major pathways that involve different PRRs-induced Type 1 INF secretion in response to hMPV infection. The first pathway involves retinoic acid-inducible gene I (RIG-1) (relevant in AEC) and melanoma differentiation-associated protein 5 (MAD5) (relevant in DC)—helicases from the RIG-I-like receptor (RLR) family [74], and the second pathway involves Toll-like receptor 3 (TLR3) and TLR7 [75].

Upon hMPV infection, the recognition of viral double-stranded RNAs (dsRNAs) and uncapped 5’-triphosphates of the viral genome triggers the coupling of RLRs with the mitochondrial antiviral signaling protein (MAVS) [74,75,76]. This activates a cascade of downstream signaling pathways leading to the secretion of Type I IFNs. Mechanistically, MAVS activates IFN regulatory factor 3 (IRF3), which mediates the secretion of Type I IFN. In addition, it also results in the secretion of proinflammatory cytokines via IRF-7 and Type III IFNs in an NF-κB-dependent manner [75]. In the second pathway, the viral products are recognized by TLRs, mainly TLR3 (in most cells) and TLR7 (in plasmacytoid DC). This leads to the activation of IRF3 and IRF7 via adaptor proteins TIR domain-containing adaptor-inducing IFN-β (TRIF) and MyD88, respectively, resulting in the secretion of Type I IFNs [75,77]. Moreover, hMPV is self-equipped with glycoprotein (G) and phosphoprotein (P) that impair viral sensing through the inhibition of RIG1 and MAVS signaling [75,76]. Mainly, TLR4 signaling is impaired by viral G protein, even though the exact mechanism behind this phenomenon is not clearly available [57,78]. Direct anti-apoptotic effects of the SH protein in primary human airway cells are suggested by one report but have not been reproduced. The key virulence factors that negatively impact the signaling mediated through PRR have been identified as M2-2 and SH proteins. These proteins interact with the MyD88 and NF-κB-adaptor proteins of TLR [79,80]. Furthermore, reports depicting the impaired upregulation of IFN-β mediated through the M2-2 interaction with MAVS are available [77]. Ren et al. reported a downregulated expression of IFN-⍺ receptors viz Jak-1 and Tyk-2, upon hMPV infection [79]. In short, a strong IFN response to HPMV in AEC is attributed to IRF3, MDA5, RIG-1, or TLR3, while in plasmacytoid dendritic cells (pDCs), it operates through the TLR7-IRF7 manner.

In addition to IFN response, Lay et al. reported a strong interleukin 33 (IL-33) and thymic stromal lymphopoietin (TSLP) expression in response to hMPV infection in the AECs using an in vitro model [15]. These cytokines induce the expression of OX40L, leading to the activation of DCs, resulting in the priming of CD4+ T cells and their differentiation to Th2 cells [81,82]. Also, these activated DCs produce a Th2-enhancing chemokine and attractor of natural killer (NK) cells, CCl7 [83,84]. Later, TSLP was identified as a key molecule in hMPV-induced lung inflammation, conducted through the genetic ablation and immunological blocking of the TSLP-TSLR pathway in hMPV-infected mice [15]. Further research conducted by another group reported that the pro-inflammatory long form of TSLP is induced by hMPV infection, while the short form remains unchanged, and this induction is attributed via NF-κB-RIG-I-TLR3 manner facilitated via TANK binding kinase 1 (TBK1) [85]. In summary, hMPV hampers or delays the efficient Th1 antiviral response through the activation of the TSLP pathway and favors the Th2 response [15].

## 6. Clinical Manifestations of hMPV Infection

Human metapneumovirus, a significant cause of respiratory tract infections (RTIs) across every age group, shows broad clinical scenarios ranging from asymptomatic to symptoms such as mild upper respiratory tract infections (URTIs) to severe pneumonia [86] (Table 1). First identified in 2001, the clinical features of an hMPV infection are similar to an RSV infection, especially in children, and remain a significant global burden, particularly in the case of infants, the elderly, and individuals with compromised immune systems [1,19,20,87,88,89].

### 6.1. Clinical Presentation in Children

hMPV infection represents a significant cause of respiratory tract infections during the first year of life [20,90]. Clinical manifestations of hMPV in children can present as both URTIs and LRTIs [2,20,91]. A study conducted at the Vanderbilt Vaccine Clinic in Nashville, USA, identified the common presentations of hMPV-associated LRTIs in children. The findings indicated that hMPV-associated LRTIs could be presented as bronchiolitis (59%), croup (18%), asthma exacerbations (14%), and pneumonia (8%). The mean age of children affected by hMPV in this study was 11.6 months [20]. Few other studies also support these symptoms of hMPV-associated LRTIs [2,3,16,92,93]. RSV-infected children exhibit more severe symptoms such as dyspnea, feeding difficulties, and hypoxemia [3,16,38]. Although not frequent, hRSV-hMPV coinfections cause more severe symptoms, higher rates of hospitalization, and greater healthcare utilization, particularly in children, the elderly, and immunocompromised individuals [94].

### 6.2. Clinical Presentation in Adults

In adults, hMPV is a common infection, and clinical features often range from asymptomatic to mild to moderate URTIs; however, it can also cause serious LRTIs that require hospital admission [33,35]. Even healthy adults present with mononucleosis-like illness [95]. In adult patients admitted to general hospitals, RSV and hMPV are linked to severe respiratory conditions that necessitate increased ventilation or oxygenation. Infection with these viruses in hospitalized patients can also cause exacerbations of asthma, COPD, and congestive heart failure (CHF) [96].

### 6.3. Clinical Presentation in Elderly and Immunocompromised

hMPV infection or reinfection contributes significantly to the burden of illness in older adults, especially immunosuppressed nursing home individuals with comorbid conditions such as chronic cardiac disease or frailty [24,97,98]. A study suggests that in recent years, most hospitalized patients with hMPV infections are elderly (mean age 75 years), and 85% have chronic heart or lung disease. Elderly people suffer from dyspnea and wheezing more compared to younger people, who manifest more hoarseness [24,98].

Immunocompromised patients, including solid organ transplant (SOT) and hematopoietic stem cell transplantation (HSCT) patients, hMPV, and other respiratory viruses, create a higher risk of morbidity and mortality when progressing from the upper to the lower respiratory tract [99,100]. hMPV is a recognized cause of both symptomatic and asymptomatic infections in lung transplant recipients. Among hematopoietic stem cell transplant (HSCT) patients, hMPV-related mortality has been reported to reach 39% [99,100,101].

### 6.4. Evidence Gaps in Clinical Research

Long-term pulmonary sequelae (wheezing, asthma) after hMPV in older children and adults are almost unstudied (virtually all data derive from early life infection). The true contribution of hMPV to COPD exacerbations and congestive heart failure decompensations in the elderly is probably underestimated because of low testing rates outside research settings. Impact of viral load, genotype (A vs. B), and co-infection on clinical severity remains conflicting across studies and requires standardized prospective cohorts [99,100,101].

## 7. Diagnosis and Surveillance of hMPV Infection

Early diagnosis of hMPV infection is crucial for implementing effective disease control measures, including outbreak prevention and timely patient care. Given the highly conserved amino acid sequences of the F protein in both hMPV and RSV, this similarity may offer valuable insights for diagnostic and therapeutic strategies [102]. Limited serological technologies have been developed to detect hMPV-specific antibodies [35,103,104,105]. Diagnosing hMPV infection can be made using several techniques, including culture, nucleic acid amplification tests (NAAT), antigen detection, and serologic testing [106].

### 7.1. Nucleic Acid Amplification Tests and hMPV Culture

The detection of viral RNA using nucleic acid amplification tests (NAATs), such as reverse transcription polymerase chain reaction (RT-PCR), is the most sensitive method for diagnosing hMPV infection [107,108,109]. However, viral culture is challenging since hMPV exhibits slow growth in conventional cell cultures and produces mild cytopathic effects. A rapid culture technique known as shell vial amplification is used to enhance detection efficiency [106].

### 7.2. Antigen Detection and Serologic Testing

Methods for detecting hMPV antigens, such as enzyme immunoassay (EIA) and enzyme-linked immunosorbent assay (ELISA), are not commonly used. No commercial immunochromatographic assays are available. A direct immunofluorescent-antibody (IFA) test, a rapid test in which labeled antibodies are used to detect specific viral antigens in direct patient materials, could be useful for diagnosing hMPV infections in outbreaks. The test results are known within two hours.

However, the sensitivity of IFA is lower than that of RT-PCR and needs to be validated before use. Detection of the immune response against the virus by serologic testing is only used for epidemiologic studies. One disadvantage of serology is that the interval between virus spreading and detection of hMPV-specific IgM and IgG antibodies is relatively long. However, a combined approach using serology and RT-PCR enhances diagnostic accuracy in hMPV infections, particularly when assessing the scale of an outbreak, such as in long-term care facilities [106,107,108,110].

### 7.3. Loop-Mediated Isothermal Amplification

Loop-mediated isothermal amplification (LAMP) technology is one of the most widely utilized isothermal methods for pathogen diagnosis [109]. LAMP is conducted in an isothermal environment (~65 °C) with high amplification efficiency, eliminating the need for complex thermal cycling equipment. Additionally, including two or three pairs of primers in the reaction system enhances specificity and amplification efficiency [111,112].

### 7.4. Recombinase-Aided Amplification and Direct Immunofluorescence Assay

Recombinase-aided amplification (RAA) assay is a recently developed isothermal amplification method characterized by simple operation, minimal equipment requirements, and high amplification efficiency [113]. The reverse transcription recombinase-aided amplification assay (RT-RAA) reaction system is conducted at 39 °C for 15 min, requiring less runtime compared to the RT-qPCR method [114]. The direct immunofluorescence assay (DFA), which employs virus-specific antibodies, is a swift method for detecting respiratory viruses and is widely utilized in diagnostic laboratories. Specific antibodies for hMPV have been commercially produced for use in DFA. However, it is important to note that this method may not achieve the same sensitivity as RT-PCR in detecting hMPV [115].

### 7.5. Metagenomic Next-Generation Sequencing

Metagenomic next-generation sequencing (mNGS) represents a cutting-edge, high-throughput diagnostic approach that is increasingly utilized for the analysis of genetic material from diverse microorganisms without prior knowledge of specific pathogens [116]. The application of nanopore metagenomic sequencing technology in exploring the nosocomial transmission of hMPV among hematology patients demonstrated notable effectiveness, successfully producing hMPV reads from 80% of the samples (20 out of 25) and retrieving complete hMPV genomes from 15 of those samples. These data indicate that this technology can be used to diagnose hMPV infection, but the sensitivity should be further improved [116]. Inclusion of hMPV in virtually all commercial multiplex panels has increased detection rates 5–10-fold in routine clinical practice. Combined serological + molecular approaches during nosocomial outbreaks achieve >98% case ascertainment [116].

## 8. Treatment and Prevention of hMPV

Although there is no specific antiviral treatment for hMPV, most current therapeutic approaches are supportive and symptomatic, prioritizing lowering respiratory symptoms and avoiding consequences [61].

### 8.1. Supportive Care

As of this moment, nearly all hMPV infection methods are supportive. Intravenous hydration and oxygen supplementation, using antipyretics such as acetaminophen or ibuprofen to control fever, and decongestants to reduce nasal symptoms are the main treatments for hospitalized infants and children. Despite extensive empirical use, bronchodilators and corticosteroids lack evidence to support their effectiveness [117]. Saline drops and nasal suctioning are often used in pediatric populations to alleviate nasal blockage. These interventions aim to ensure patient comfort while the immune system resolves the infection.

Oxygen supplementation is essential in hospitalized patients, particularly those with hypoxemia or respiratory distress. Non-invasive positive pressure ventilation and high-flow nasal cannula oxygen therapy are frequently used [118] to treat acute respiratory distress. In severe cases, especially in newborns and elderly individuals with comorbidities, mechanical ventilation may be required [119].

### 8.2. Antiviral Therapies

Even though hMPV is a widespread issue, there are presently no approved antiviral medications to treat it. However, preclinical research and small-scale clinical trials have investigated several potential antivirals. The broad-spectrum antiviral ribavirin (anecdotal use only) has demonstrated in vitro action against hMPV, with varying degrees of success in animal models [61]. Due to serious side effects such as hemolytic anemia and teratogenicity, its clinical use is restricted. It is usually saved for severe infections in immunocompromised adults, such as lung transplants, cancer, stem cell transplants, or people infected with Middle East respiratory syndrome coronavirus (MERS-CoV) [120].

For severely immunocompromised patients with progressive lower-respiratory-tract disease, ribavirin ± IVIG (intravenous immunoglobulin) is sometimes used on a compassionate basis, but this practice rests on case reports and small retrospective series only; no controlled evidence of efficacy exists. The most promising near-term specific interventions are anti-F monoclonal antibodies and combined hMPV/RSV vaccines now in clinical development, but none are available for clinical use today. In in vitro and animal models, monoclonal antibodies have demonstrated promising results targeting the hMPV F protein, promoting viral fusion and entry. Passive immunity in the form of neutralizing antibodies is an additional alternative for prophylaxis or treatment in the absence of an approved vaccine. Even though these treatments have not been implemented in clinical settings yet, research is still being conducted to develop them for both preventative and therapeutic purposes [121].

### 8.3. Immunomodulatory Therapies

Studies comparing vaccinated and experimentally infected mice have shown that the acquired immune response triggered by hMPV is unable to effectively remove the virus from the airways, resulting in lung damage and an exaggerated inflammatory response. Poor T and B cell immunological memory development occurs after disease clearance, which is thought to encourage reinfections and viral dissemination in the population [56,122].

Corticosteroids and other immunomodulatory drugs have been attempted to reduce inflammation, however, their use is still debatable. Although some research indicates that corticosteroids may improve oxygenation and lessen lung inflammation, other trials show no discernible therapeutic effect or perhaps a higher risk of subsequent infections. Systemic corticosteroids are not routinely recommended for hMPV infection. Their use should be restricted to patients with wheezing due to underlying asthma or COPD exacerbation triggered by hMPV.

Corticosteroids are, therefore, not usually advised for hMPV infections unless there are exacerbations of COPD or asthma as concurrent diseases [123].

### 8.4. Adjunctive Therapies

For hMPV, adjuvant medicines such as immunoglobulin therapy and interferon-based treatments have been researched. Pediatric patients with hMPV infection who are immunocompromised had elevated mortality and LRTI rates. More research is needed to determine the advantages of ribavirin and intravenous immunoglobulin treatment in this patient population [124]. However, when IFN-λ was administered to hMPV-infected mice, the lung inflammatory response, viral titer, and disease severity decreased. The hMPV G protein was also involved in controlling the virus-induced IFN-λ response. These findings demonstrate that type III IFNs, or IFN-λs, are essential for protection against hMPV infection [125].

## 9. Vaccine Development and Future Prospects

No vaccine development for hMPV has progressed significantly in recent years, and no vaccine is currently licensed for clinical use. Multiple platforms are under investigation, including live-attenuated viruses, recombinant viral vectors, subunit vaccines (primarily based on the pre- or post-fusion F protein), virus-like particles, and mRNA-based approaches [126,127,128,129]. Among these, chimeric live-attenuated candidates and prefusion-stabilized F-protein subunit vaccines have shown the most promising immunogenicity and protection in preclinical animal models and early phase human trials. A bivalent hMPV/RSV prefusion F vaccine candidate (mRNA-136) and a virus-like particle-based candidate (IVX-A12) are currently being evaluated in phase 1/2 clinical studies. Monoclonal antibodies targeting the hMPV F protein have demonstrated prophylactic and therapeutic efficacy in animal models and are also advancing toward clinical evaluation. Although these candidates offer hope for future prevention, particularly in high-risk populations, effective outbreak control through vaccination is not yet possible. Although the most promising vaccine approach for hMPV is a live attenuated vaccine, there is currently no approved vaccine against it. However, finding an attenuated virus strain with the optimal balance of immunogenicity and attenuation is still challenging. Reverse genetics have been used to create a panel of recombinant hMPVs that were specifically defective in ribose 2′-O methyltransferase (Mtase) but not G-N-7 Mtase. Despite their high immunogenicity, these Mtase-defective hMPVs were genetically stable and adequately attenuated [127,128]. Subunit vaccines based on the F protein are also being studied for their potential to induce neutralizing antibodies [129].

If successful, the availability of effective vaccines will significantly reduce the reliance on supportive care and therapeutic interventions, especially in high-risk populations, and may enable the long-term control of hMPV circulation. To date, no hMPV vaccine has progressed beyond phase 2 clinical trials.

## 10. Conclusions

Human metapneumovirus remains a major global cause of acute respiratory illness, particularly in young children, older adults, and immunocompromised individuals. Despite two decades of research, important knowledge and clinical gaps persist: (1) the true disease burden in low- and middle-income countries and in adults remains underestimated because of limited systematic testing; (2) no specific antiviral drug or licensed vaccine is available; (3) the immunological basis for frequent reinfection and the link between early life hMPV infection and subsequent wheezing/asthma requires further elucidation; and (4) rapid, affordable point-of-care diagnostics suitable for resource-limited settings are still lacking. Promising directions include prefusion-stabilized F-protein subunit and mRNA vaccines (currently in phase 1/2 trials), long-acting monoclonal antibodies, and novel small-molecule fusion inhibitors. Large-scale phase 3 efficacy trials improved global surveillance, and studies of combination hMPV/RSV vaccines will be critical to reduce the substantial morbidity caused by this pathogen in vulnerable populations worldwide. Key priorities for the coming years include: (i) completion of phase 2/3 trials of the most advanced candidates (notably the bivalent hMPV/RSV prefusion F vaccine IVX-A12 and mRNA-based platforms), (ii) evaluation of monoclonal antibodies targeting the hMPV fusion protein for passive immunization of high-risk infants and immunocompromised individuals, (iii) deeper elucidation of incomplete natural immunity and viral immune-evasion mechanisms to guide next-generation vaccine design, and (iv) establishment of systematic global surveillance, particularly in currently underrepresented regions such as Latin America and Southeast Asia, to better define disease burden and circulating genotypes. Large-scale clinical trials, long-term epidemiological research, and improved worldwide surveillance are crucial for reducing the public health impact of hMPV. To lessen the burden of hMPV on vulnerable populations worldwide, considerable progress can be made in creating efficient preventive and therapeutic techniques by tackling these issues.

## Figures and Tables

**Table 1:** Signs and symptoms of hMPV infection. Abbreviation: Chronic obstructive pulmonary disease (COPD), Congestive heart failure (CHF), Lower respiratory tract infection (LRTI), Solid organ transplant (SOT), and Hematopoietic stem cell transplantation (HSCT).

Population	Common Symptoms	Severe Cases	Risk of Hospital Admission
Children	Fever, cough, coryza, rhinorrhea, hoarseness, otalgia, rhinitis, conjunctivitis, pharyngitis, abnormal tympanic membranes	Bronchiolitis, croup, asthma exacerbations, pneumonia	Higher in infants, especially severe LRTI cases
Adults	Respiratory distress, coughing, nasal congestion, fever, conjunctivitis, otitis media, wheezing, pneumonia, bronchitis, sore throat	Exacerbations of asthma, COPD, CHF, severe LRTI requiring hospitalization	Severe LRTI, asthma/COPD exacerbations
Elderly	Influenza-like symptoms, fever, cough, dyspnea, fatigue, pneumonia, pneumonitis	Higher rates of pneumonitis, severe LRTI, higher mortality in hospitalized cases	Chronic heart/lung disease
Immunocompromised	Rhinorrhea, cough, sputum production, severe pneumonia, acute graft dysfunction, acute respiratory distress syndrome, graft rejection	Higher mortality in SOT/HSCT patients, graft rejection, fatal pneumonia	Severe cases lead to hospitalization, high mortality in transplant recipients

## Data Availability

Not applicable.
